# Supplementary figures and images for: Gain efficiency with streamlined and automated data processing: Examples from high-throughput monoclonal antibody production
Source: PLoS One. 2025 Jul 1;20(7):e0326678. doi: 10.1371/journal.pone.0326678 (PMC12212921; doi:10.1371/journal.pone.0326678)

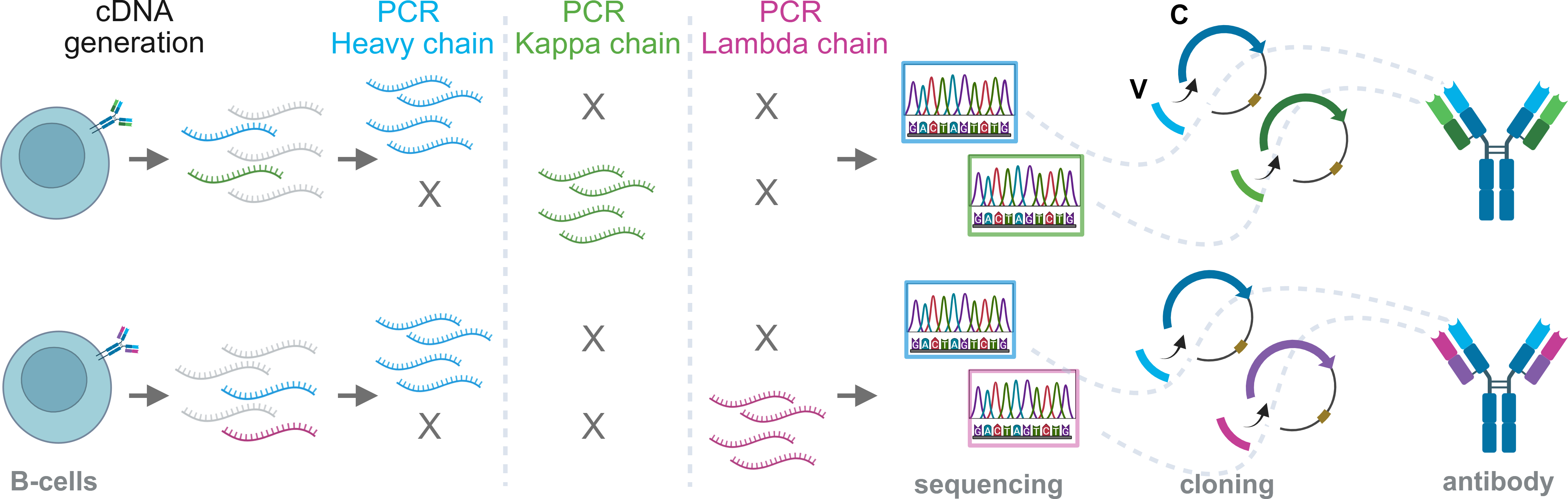

Supplement: S1 Fig — After cDNA generation, three parallel PCR reactions are performed to amplify the heavy chain variable region (light blue) and either the Kappa (light green) or Lambda (pink) variable region. Upon verification by sequencing, the variable regions (V) are cloned into plasmids encoding the constant part (C) of the respective antibody chain (dark blue: heavy constant part; dark green: Kappa light constant part; purple: Lambda light constant part). See Fig 2 for a comprehensive overview. For an overview on B-cell receptor and antibody variability, refer to Fig 1 by Khatri et al. [51] and Fig 1 by Mikocziova et al. [52]. (DOCX) [file pone.0326678.s001.tif]

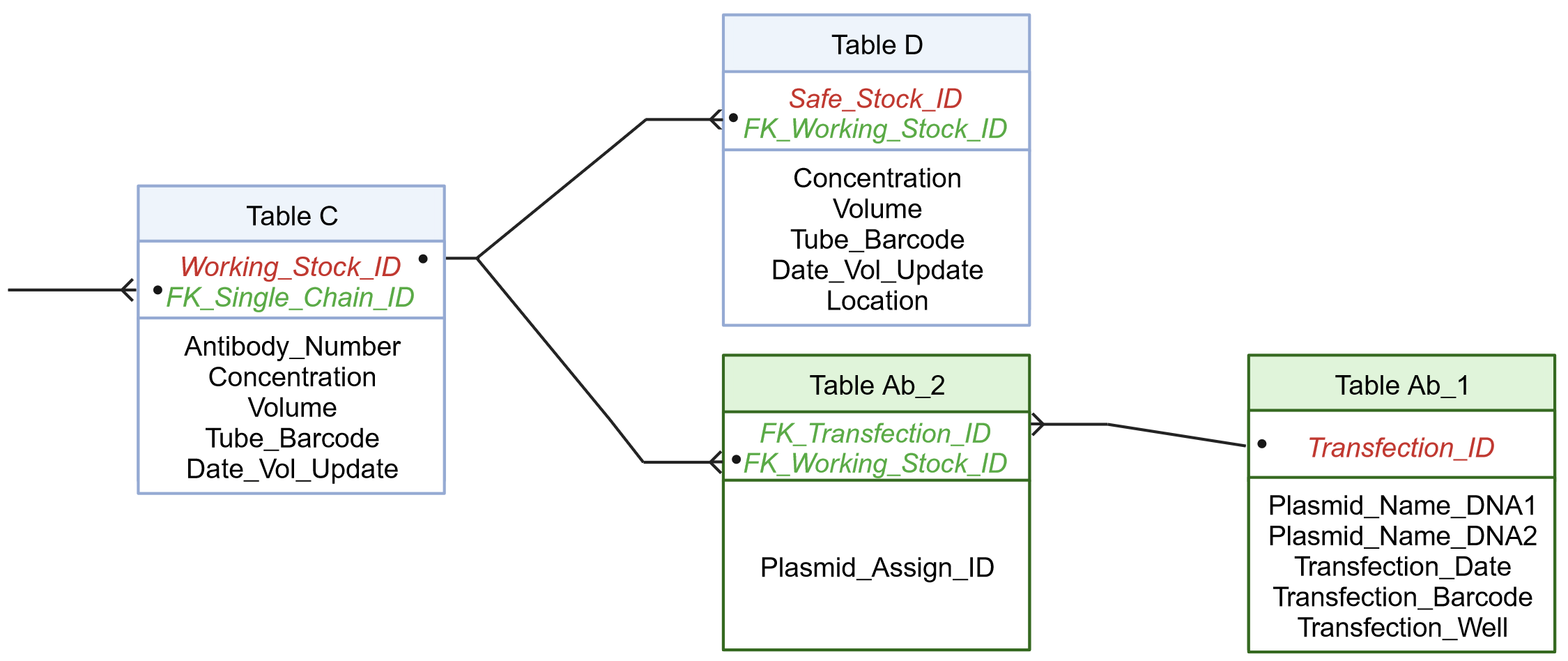

Supplement: S2 Fig — Table C stores information on isolated plasmids of paired antibody chains (heavy and light), and Table D is a repository of plasmid aliquots stored as safe stocks in separate (physical) locations for future reproduction of plasmids or downstream applications. Table Ab_2 connects the information on plasmid pairs with subsequent transfection of HEK cells. Fields (i.e., columns) Working_Stock_ID, Safe_Stock_ID and Transfection_ID store unique IDs of each record per table. Fields starting with FK_ are records’ IDs inherited from related tables. Records (sample states) are connected through one-to-many relationships in the database structure; for example, a plasmid from Table C can be used for creation of a safe stock aliquot (in Table D) multiple times, while one aliquot of safe stock (in Table D) is associated with exactly one plasmid from Table C. Similarly, one transfected HEK cell sample (i.e., transfection well, in Table Ab_1) is associated with exactly two plasmids (a pair of heavy and light chain plasmids, in Table C), while this plasmid pair can be used for multiple transfections. (DOCX) [file pone.0326678.s002.tif]

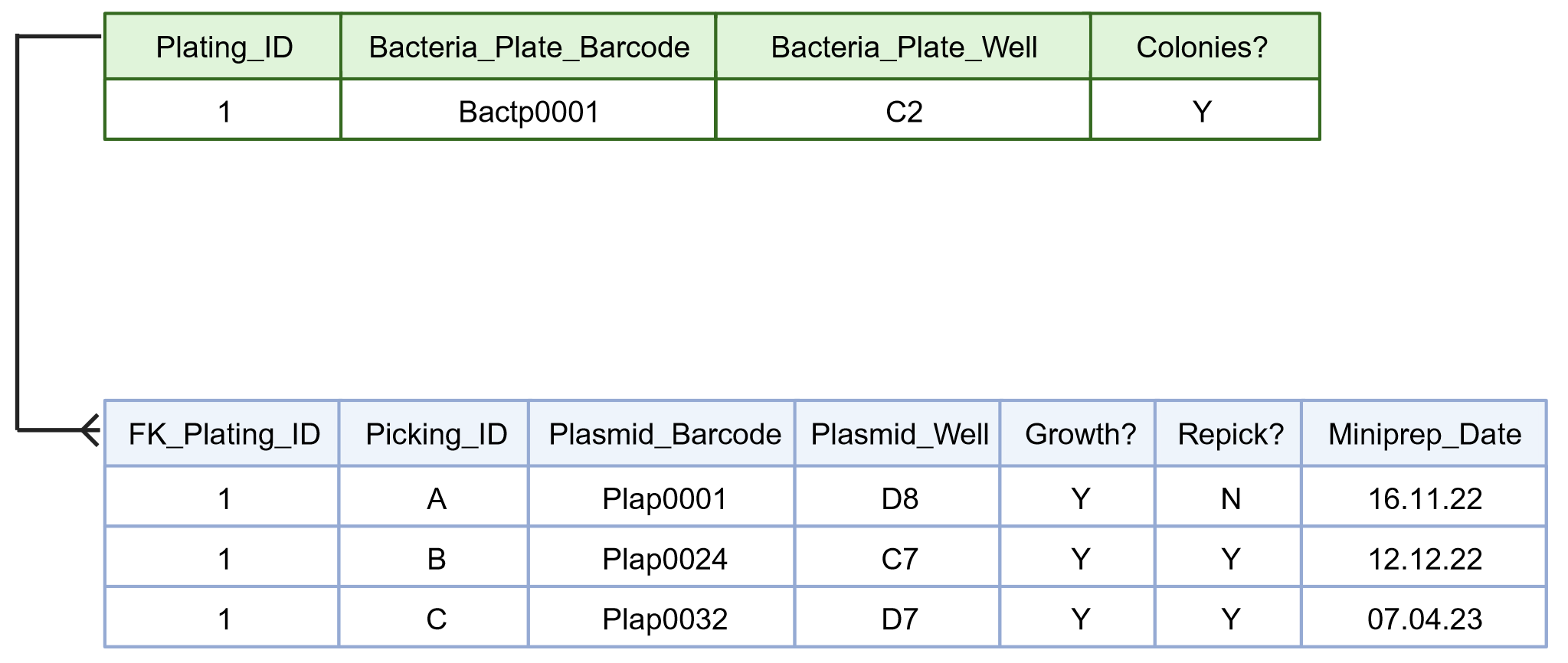

Supplement: S3 Fig — Picked bacterial colonies can be connected to already imported bacteria plate information through the identifiers (Plating_ID/FK_Plating_ID), reducing the redundancy of stored data. The identifier Plating_ID is unique in the bacteria plating table but not in the colony picking table, allowing for picking of multiple colonies from the same bacteria plate (one-to-many relationship in the database structure). The colony picking table has its own unique identifier (Picking_ID). (DOCX) [file pone.0326678.s003.tif]

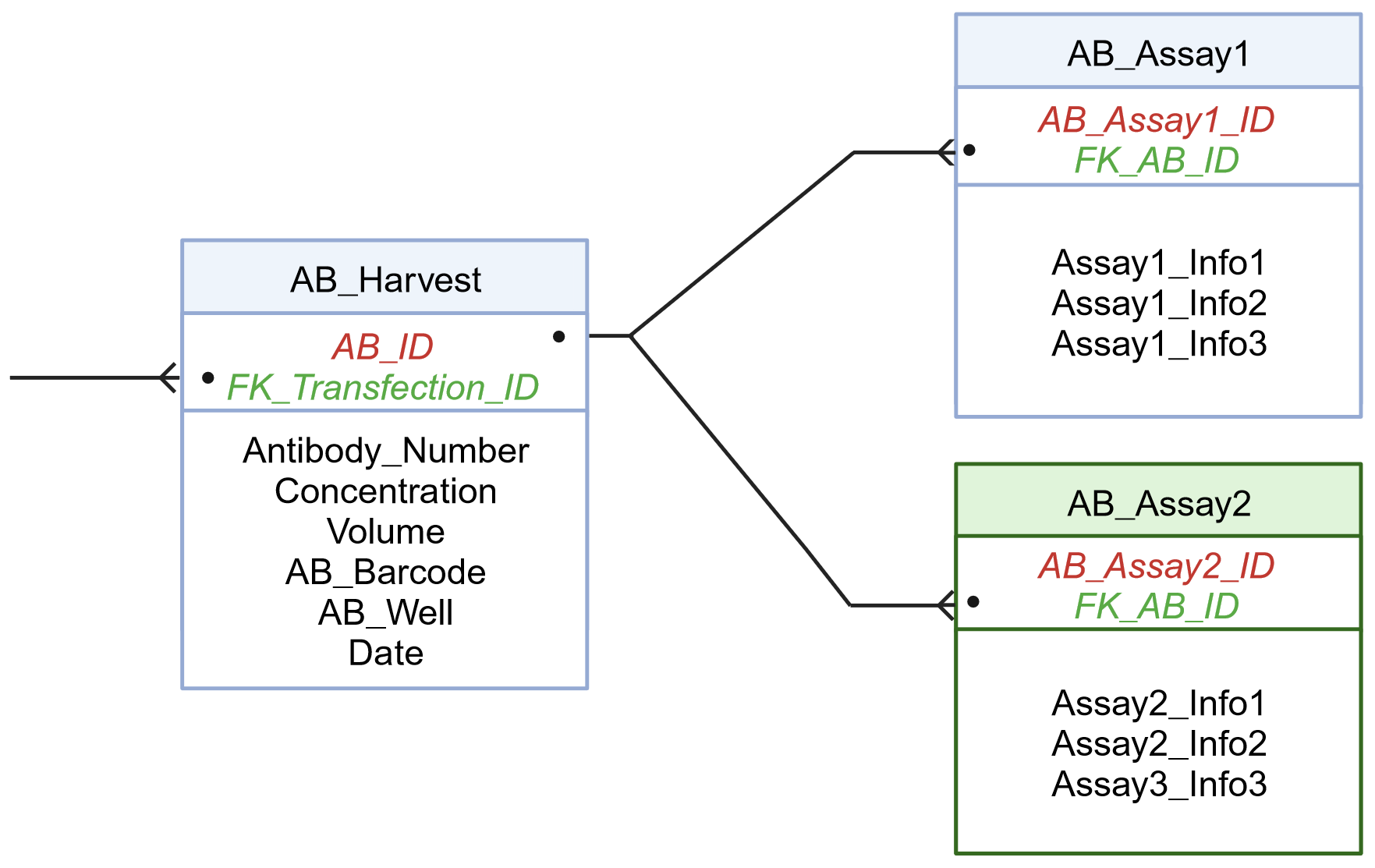

Supplement: S4 Fig — The flexibility of the database design enables smooth integration of new information (e.g., experimental readouts), which allows for efficient management of diverse datasets within the database. Here, the information on two hypothetical functional antibody assays (AB_Assay1 and AB_Assay2) are appended to the information on the harvested antibody through the AB_ID identifier, thus linking to any previous information on that sample (starting from the B-cell donor/patient). (DOCX) [file pone.0326678.s004.tif]

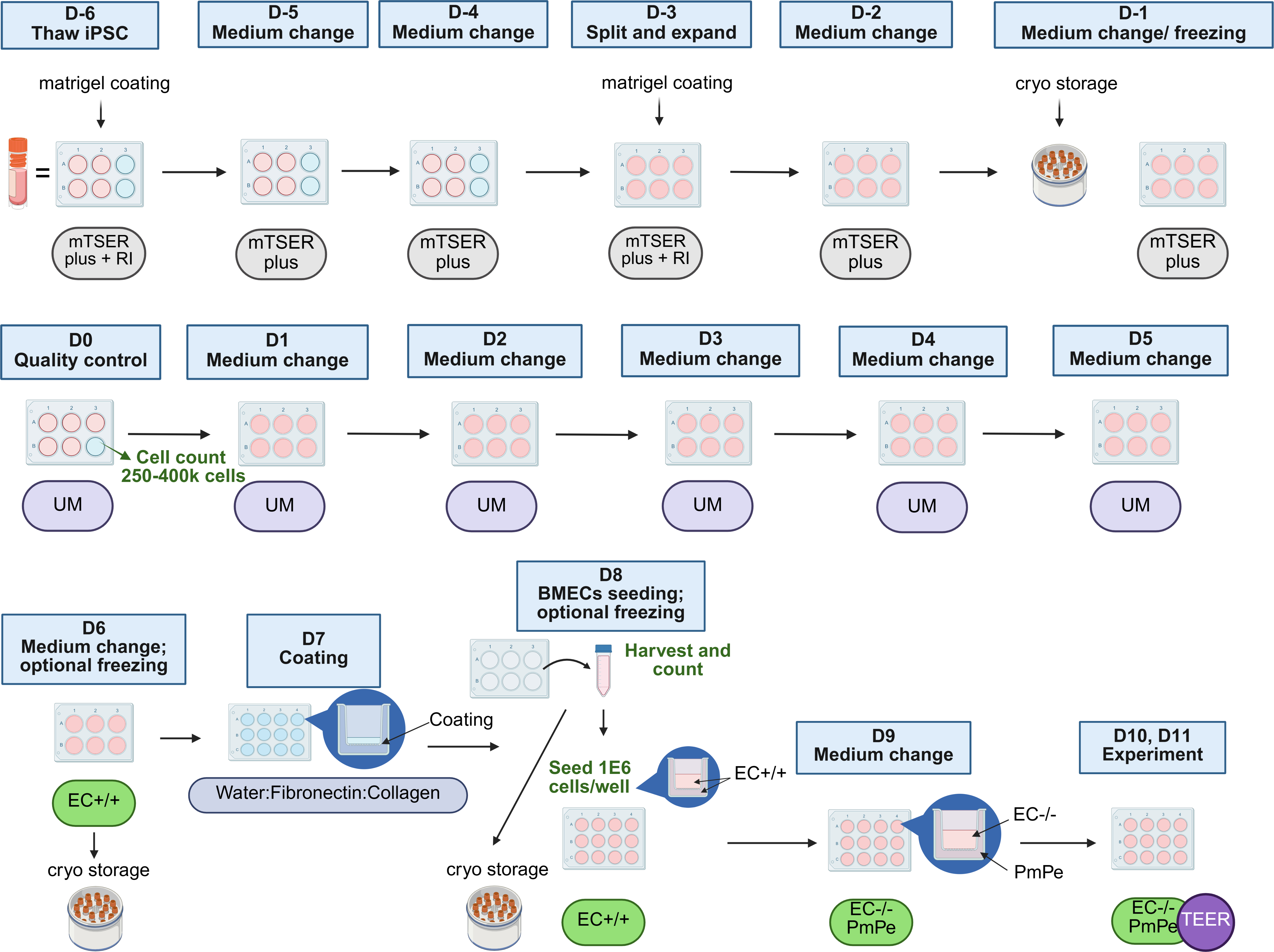

Supplement: S5 Fig — Wet lab steps and timepoints (relative to the start of the differentiation – day 0: D0) are indicated by blue rectangles. Cycles of thawing (D-6), possible freezing (D-1, D6, D8), harvest and seeding (D-3, D8) of cells. Count and viability assays are carried out on D-3, D0 and D8. Media used at each timepoint are indicated by ellipses – gray: mTSER plus medium with/without rock inhibitor (RI); violet: Unconditioned Medium (UM); green: Endothelial Cell Medium with/without supplements (EC + / + , EC -/-, respectively). The TEER measurement timepoints (D10, D11) are indicated by a purple circle. For a detailed protocol, refer to Fengler et al. [34]. (DOCX) [file pone.0326678.s005.tif]

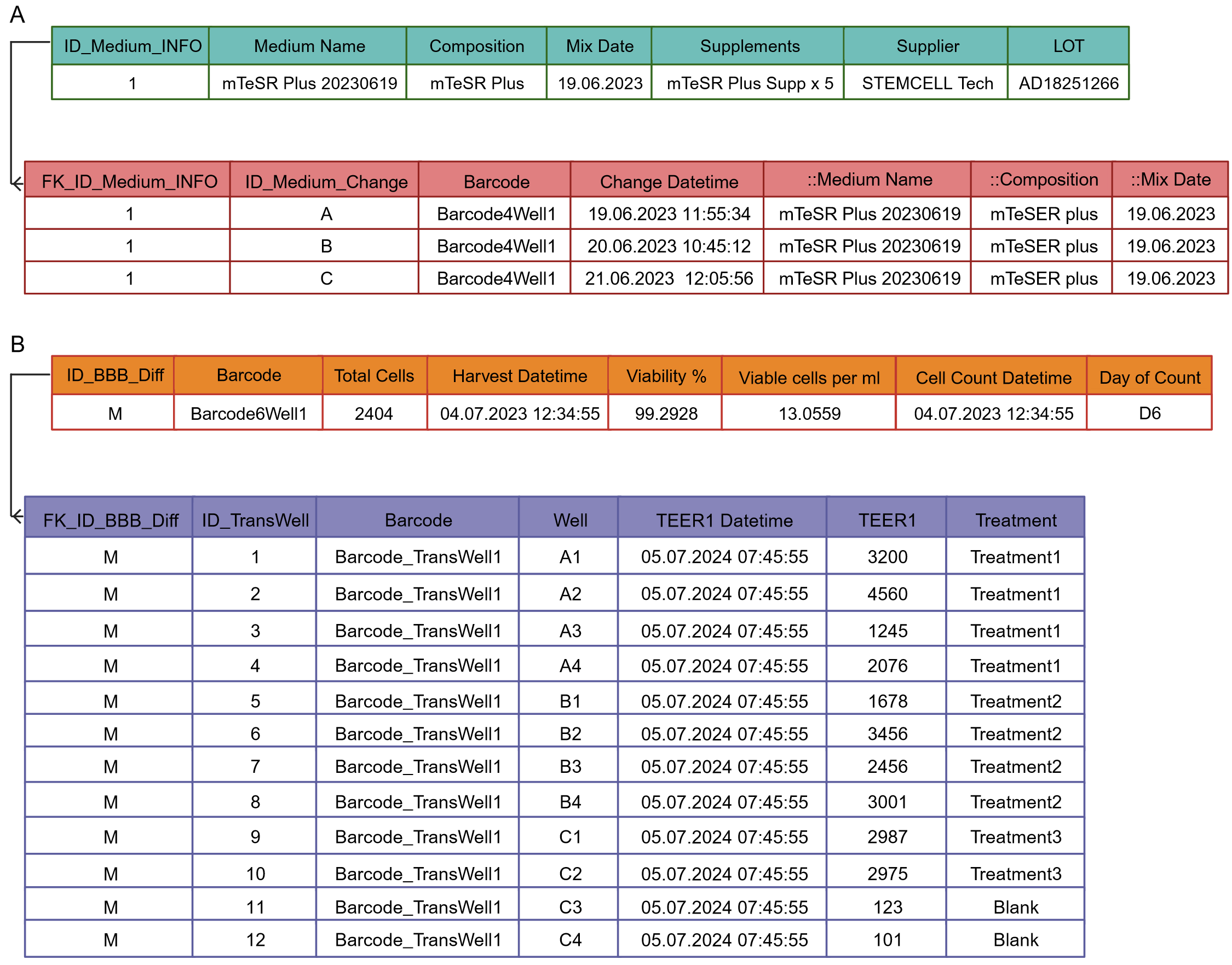

Supplement: S6 Fig — A) No-redundancy principle. The information on each medium batch is stored in the database only once (upper table, green headers). Whenever a media change is performed, the unique identifier of the concerned medium batch (ID_medium_INFO) is fetched on the backend by a File Maker script and populated in the media change table (lower table, red headers). Information in columns::Medium Name,::Composition, and::Mix Date is fetched from the medium table (upper table, green headers). Information in the column::Barcode is fetched from another table (not shown). B) Example of the harvest event that guided the design of database structure: pulling differentiated cells from 6-well plate and seeding on 12-well TransWell plate. At this workflow stage, cells could also be cryo-stored for future experiments. Implementing a one-to-many relationship between 6-well and 12-well TransWell plates helps avoid storing redundant information. (DOCX) [file pone.0326678.s006.tif]

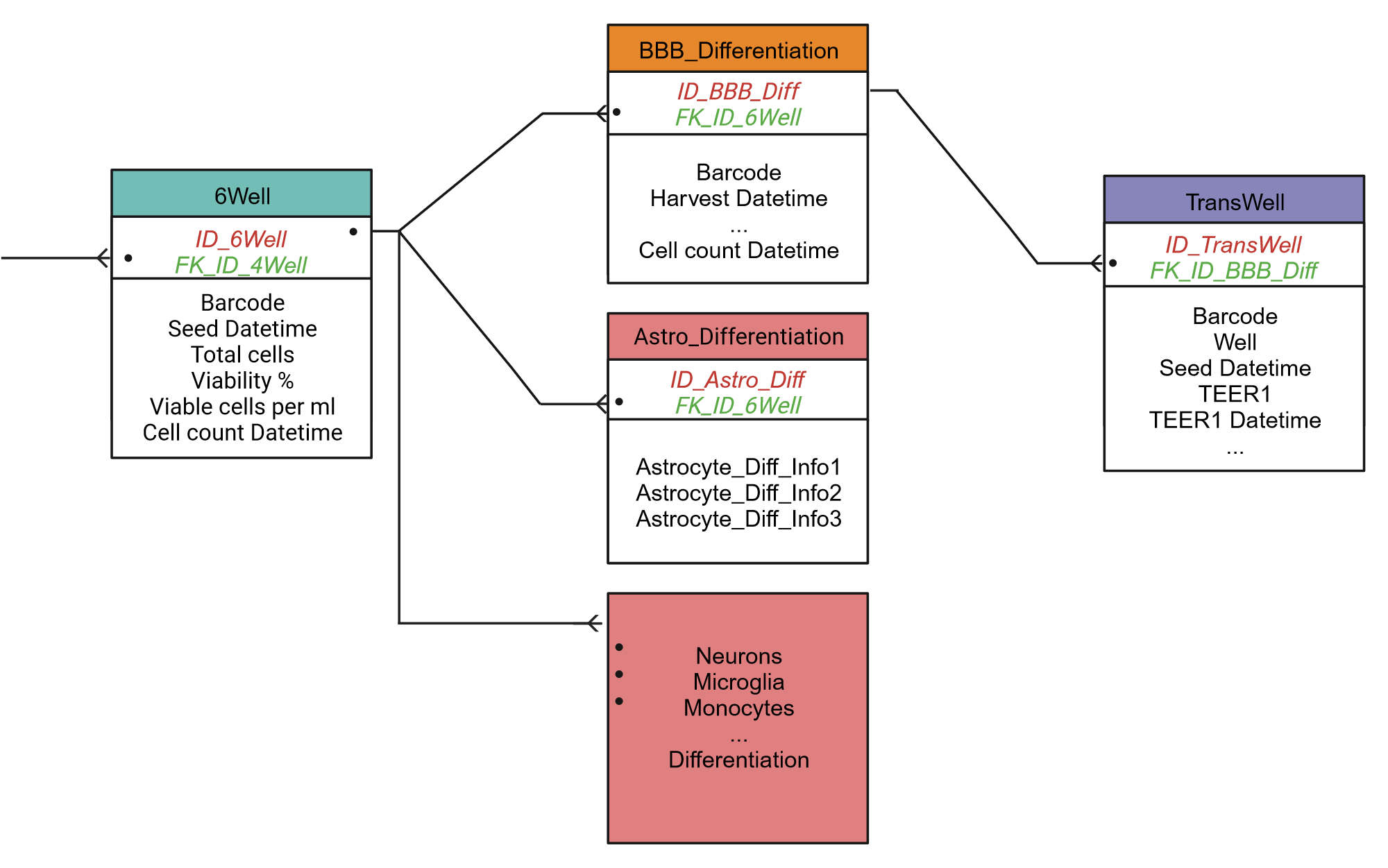

Supplement: S7 Fig — Possible expansion of database structure towards differentiation of other cell types (such as astrocytes, neurons, microglia, monocytes; red tables). The structure is flexible and allows appending of tables to a 6-well plate table (green) that stores information on undifferentiated cells through a one-to-many relationship. In this way, different differentiation protocols can easily be accommodated in the database. (DOCX) [file pone.0326678.s007.tif]
