# Supplementary material for: Gain efficiency with streamlined and automated data processing: Examples from high-throughput monoclonal antibody production
Source: PLoS One. 2025 Jul 1;20(7):e0326678. doi: 10.1371/journal.pone.0326678 (PMC12212921; doi:10.1371/journal.pone.0326678)
Supplement: Attachment — Submitted filename: REV2_rebuttal Letter.docx [file pone.0326678.s011.docx]

Dear Dr. Bhanwar Lal Puniya, Dear editors and reviewers,

We sincerely thank you for your evaluation of our manuscript, “Gain Efficiency with Streamlined and Automated Data Processing: Examples from High-Throughput Monoclonal Antibody Production.” We highly appreciate the reviewers’ constructive comments, which have helped us improve the quality of our work. We have carefully addressed all the concerns raised and believe that the revisions have further strengthened the manuscript. Thank you once again for your time and effort in reviewing our submission.

In the following, we list all comments and detail how we addressed them.

Journal requirements:

Authors are advised to revise the manuscript according to the comments provided by Reviewer #4.

We have addressed all comments and suggestions provided by Reviewer #4. We are thankful for the critical review process and hope that the improved manuscript now fully meets the publication criteria of PLOS ONE.

Reviewer #4:

I would like to thank the authors for their efforts in preparing this manuscript. It presents an important contribution, please addressed below comments:

We thank Reviewer #4 for the critical evaluation of our manuscript and for recognizing its importance. We have addressed all comments in the revised version and expanded on those in our rebuttal letter.

1. Some sections feel a bit dense and could be made clearer by breaking up longer sentences and simplifying complex descriptions. This would help improve readability and accessibility, especially for readers who may not be familiar with all the technical details. (answered together with comment no. 2, below comment no. 2)

2. The transitions between sections, particularly from general workflow principles to specific implementation details, could be smoother. A clearer connection between these parts would help guide the reader more effectively.

We appreciate these valuable suggestions. In response, we have revised the main text by breaking up longer sentences and making technical details easier to follow and read. Complex descriptions have been simplified where appropriate, without compromising the essence and structure of the manuscript. We have also tried to establish clear links between general and technical details, especially in our ‘Examples from the workflow’ sections. This also helps to follow the logical structure of the manuscript better. Our Glossary (Box 1) further aids readers at the start by defining data processing concepts often used throughout the text (refer to page 5) and was also refined to convey clearer messages in shorter sentences.

We think that the manuscript is now much more coherent and accessible.

3. Figure 2 seems to be an important part of explaining the workflow, but it’s not included for review. Make sure it’s well-labeled and clearly conveys the intended message.

Figure 2 is indeed an essential part of our manuscript, illustrating the entire mAB production pipeline and the many different steps involved in a logical order. Figure 2 has been recalled multiple times throughout the text, pointing out examples of how our workflow is designed. We have made sure that the figure is explained in the manuscript and that it is properly referred to in each section where appropriate. We have also labelled and annotated it. We will of course include it with our revision file (see also below).

**Fig 2**. **Workflow for the production of mABs from patient-derived individual B-cells.** Wet lab work steps (ellipse-icons with headers) and data processing steps (yellow icons). Sample flow is depicted by blue arrows and information flow is depicted by yellow arrows. The experimental procedure starts with FACS sorting of individual B-cells. cDNA is generated from individual B-cells, and PCR1 and PCR2 serve to amplify antibody chains. Successfully amplified chains, evaluated by capillary electrophoresis (cELE1), are sequenced (SEQ1). Upon positive sequence evaluation with the aBASE software [23], specific primers are used for the final amplification of the specificity-determining chain parts (PCR3). Amplification is quality-controlled by electrophoresis (cELE2). The specificity-determining regions are cloned by Gibson Assembly (GiAs) into plasmids encoding the constant parts of the chains. Plasmids are amplified by transformed (TRFO) bacteria after plating (PLA) and picking (PICK) individual bacterial clones. Prior to quality control by sequencing (SEQ2), amplified plasmids are isolated (MINI), and plasmid concentration is measured and adjusted (ConcAdj). Based on sequencing results, functional plasmids (PLA) are sorted. Matching plasmid pairs (mAB Pla) are prepared for transfection (Traf) into HEK cells. mABs produced and secreted by HEK cells are harvested (mABs), and mAB concentration is quantified (quant). Glycerol stocks (Glyc) of transformed bacteria and plasmid aliquots (Pla safe) serve as safe stocks of plasmids. The colors of the wet lab work step captions indicate different workflow sections: blue – molecular biology; red – microbiology; green – cell biology. The colors of the data processing circle contours and font indicate the utilized technology: red – Python; yellow – Knime; blue – third-party software.

4. It would also help to refer to workflow diagrams more explicitly in the text so readers can follow along more easily.

We agree with this comment and have made sure that each workflow diagram is explicitly referred to at relevant points in-text, helping guide readers through the process more clearly.

5. The manuscript briefly mentions why Python and Knime were chosen, but it would be useful to provide a stronger rationale. A short comparison of the benefits and trade-offs of these tools versus other available options would add clarity.

Thank you for the suggestion. We have expanded upon the explanation of our choice of Python and KNIME, emphasizing their complementary advantages (please see also our answer to comment no. 6 below)

6. Similarly, when discussing GUI-based vs. scripting-based approaches, consider touching on usability, performance, and reproducibility. This would give readers a better understanding of the reason behind these choices.

We appreciate the suggestion to elaborate further on our selection of tools, GUI-based and script-based approaches.

We have added a brief description of why Knime and Python were chosen, as well as some suggestions for alternative technologies (refer to “Examples from the workflow” on page 11). We have also expanded on the usage of GUI-based workflows (page 10).

Knime was chosen as a technology mainly due to its wide application and open-source system. Knime has an intuitive drag-and-drop graphical interface and can easily be used by team members without coding experience. Furthermore, workflows can be shared, ensuring reproducibility and flexibility. Knime can also embed Python scripts.

Python is very widespread in the field of data processing and bioinformatics. It is suitable for many different projects and computations. It can run on various operating systems and supports notebook-style reporting, further enhancing usability and reproducibility of workflows. The syntax used by Python is generally clean and readable, which is helpful when working with interdisciplinary teams.

The use of GUI tools in our workflow is essential to allow seamless and faster integration of researchers with limited coding knowledge on an existing pipeline. The combined use of tools allows interdisciplinary work and ensures consistency, standardized documentation, and automation of repetitive tasks.

7. The paper mentions input and output file formats but doesn’t fully address the importance of standardization for interoperability. A brief mention of commonly used formats (e.g., CSV, JSON, XML) and their advantages would help strengthen this section.

We agree that the use of standardized files plays a key role in interoperability and reproducibility across platforms. Our pipeline supports CSV files which are commonly used in laboratory settings. CSV files are easy to read, simple and compatible with spreadsheet software and most programming environments. We have clarified the use of CSV files in our manuscript (page 15). Formats such as JSON or XML support much larger nested data and metadata which we do not handle in this pipeline. Our datasets are primarily tabular, as are most datasets generated in labs. Nevertheless, we added a brief section on the context in which JSON and XML data formats could be used.

8. It would also be helpful to explain how intermediate files are validated to ensure data integrity as information moves between pipeline modules.

Thank you for the comment. Indeed, this section was lacking clarification of that point. We have added a description of our built-in validation steps for intermediate files, please refer to the box “Examples from the workflow” in the section “Defining input and output files”.

9. The discussion on computational resources (e.g., NAS, cloud services, institutional servers) could benefit from a short mention of data security and access control measures. This would be especially relevant for handling sensitive or large-scale datasets.

Thank you for the comment. We have expanded the discussion on computational resources to emphasize that, when using storage drives and servers such as NAS, cloud services, or institutional infrastructure, appropriate data security and access control measures should be in place—particularly when managing sensitive or large-scale datasets. This includes implementing practices such as user authentication, encryption, and compliance with institutional or legal data protection guidelines.

10. Since working with large datasets can present challenges, consider addressing potential issues like memory constraints or options for parallel computing.

Thank you for the suggestion, we do agree it is important to address these, especially considering ever-increasing volume of (biological) data. We have added a sentence acknowledging the challenges of working with large datasets and briefly discussed strategies such as memory management and parallel processing. Please refer to the section” Allocating separate computational space to modules” on page 14 for revision of these changes.

We look forward to your response.

Best Regards,

Dominik Stappert
